# Governance of research consortia: challenges of implementing Responsible Research and Innovation within Europe

**DOI:** 10.1186/s40504-020-00109-z

**Published:** 2020-11-16

**Authors:** Michael Morrison, Miranda Mourby, Heather Gowans, Sarah Coy, Jane Kaye

**Affiliations:** 1 Centre for Health, Law, and Emerging Technologies (HeLEX), Faculty of Law, University of Oxford, Oxford, UK; 2 Melbourne Law School, University of Melbourne, Melbourne, Australia

**Keywords:** Governance, Responsible Research and Innovation (RRI), Stakeholders, Data sharing, Public engagement, Consortia, Transparency

## Abstract

Responsible Research and Innovation (‘RRI’) is a cross-cutting priority for scientific research in the European Union and beyond. This paper considers whether the way such research is organised and delivered lends itself to the aims of RRI. We focus particularly on international consortia, which have emerged as a common model to organise large-scale, multi-disciplinary research in contemporary biomedical science. Typically, these consortia operate through fixed-term contracts, and employ governance frameworks consisting of reasonably standard, modular components such as management committees, advisory boards, and data access committees, to co-ordinate the activities of partner institutions and align them with funding agency priorities. These have advantages for organisation and management of the research, but can actively inhibit researchers seeking to implement RRI activities. Conventional consortia governance structures pose specific problems for meaningful public and participant involvement, data sharing, transparency, and ‘legacy’ planning to deal with societal commitments that persist beyond the duration of the original project. In particular, the ‘upstream’ negotiation of contractual terms between funders and the institutions employing researchers can undermine the ability for those researchers to subsequently make decisions about data, or participant remuneration, or indeed what happens to consortia outputs after the project is finished, and can inhibit attempts to make project activities and goals responsive to input from ongoing dialogue with various stakeholders. Having explored these challenges, we make some recommendations for alternative consortia governance structures to better support RRI in future.

## Introduction

**Fig. 1:**
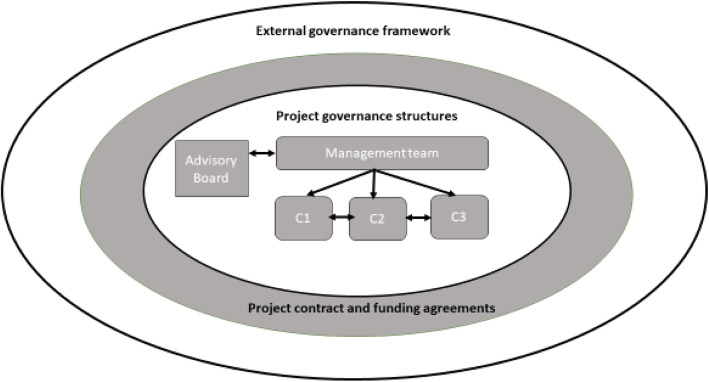
A generic governance structure for consortia (adapted from Kaye et al. 2015)

The image of the lone scientist, or even single research team, working in isolation is largely anachronistic. Scientific research in the life sciences increasingly operates through large, international consortia. This is partly driven by the move towards a more data-intensive biology and biomedicine (Leonelli 2012; Vermeulen 2009). For the purposes of this paper, we define consortia as time-limited collective research endeavours, which operate under one or more contractual agreements, and typically have a formal management structure and governance structure (see Fig. 1). This approach is exemplified by the European Union ‘Framework’ and later ‘Horizon’ science funding programmes. The core argument of this paper is that the governance arrangements that create and define consortia, especially their formal legal and contractual responsibilities, can undermine the ability of scientists to implement viable responsible research and innovation measures. The aim of this paper is to provide a detailed account of four key impediments to RRI posed by current governance arrangements, and to explicate their causes with a view to opening these issues up for wider discussion and debate.

The idea of Responsible Research and Innovation (or ‘RRI’) has been advanced to make research more responsive to broader societal concerns and needs. Although RRI originated in EU policy circles, it is of increasing global relevance as a locus of thought around responsible innovation (Gao et al. 2019). RRI includes components such as public engagement, open access, gender equality, science education, ethics, and governance. Each of these components aims, in different ways, to increase transparency, diversity, inclusiveness and adaption to change^1^ by fostering greater interaction between researchers and other stakeholders, including potential end users of new technologies and the wider communities in which they are embedded. Consortia, including public-private partnerships, should not be exempt from the outreach and engagement activities mandated by RRI. However, the structures inherited from funders, particularly in the form of standard contractual agreements which are then modified primarily by institutional representatives, are not necessarily set up with the open and externally responsive aims of RRI in mind.

This study draws on the authors’ collective experience as interdisciplinary members of a substantial number of life sciences research consortia, and in particular our knowledge of the contractual clauses and governance arrangements (e.g. Kaye et al. 2015; Morrison et al. 2015; Morrison 2017; Muddyman et al. 2013; Teare et al. 2018). The focus is necessarily European, both in terms of the definition of ‘consortia’ to reflect the norms of EU projects; and in terms of the interactions between project governance and legal instruments such as the General Data Protection Regulation (GDPR). However, the overall aim of externally-responsive innovation, and its compatibility with the internal bureaucracies of scientific research, may well be of interest outside an EU context.

Based on our assessment of the common contractual and governance arrangements in European consortia we delineate four key governance challenges of implementing RRI which are commonly encountered within large research consortia. Addressing these challenges from the outset, beginning with the contractual negotiations to establish the consortium, is (we suggest) integral to the successful implementation of RRI. We consider the aims of RRI alongside the requirements of the GDPR, which also originates in the EU but has global ramifications in its benchmarking influence and extraterritorial reach (Dove 2019). The GDPR is therefore relevant for research using human-derived data (‘personal data’) in many different international contexts; representing a high watermark of legal protection for personal data.

This paper aims to help those seeking to implement RRI in large research consortia, firstly by critically reflecting on why research consortia governance is needed and the way it is conventionally arranged. Secondly, by outlining the challenges we have identified within these conventional arrangements: contractual foundations, participant involvement, effective transparency and preparing for the project legacy; together with corresponding recommendations for meeting those challenges.

## What is responsible research and innovation (RRI)?

RRI has been influentially characterised by Owen and colleagues as a policy aim both laudable and uncontroversial at high level (few, they point out, would advocate for *irresponsible* research), but requiring more detailed exposition for it to be of implementable, practical value (Owen et al. 2012). Since then, the authors have developed a framework for the related, although distinct (Owen and Pansera 2019) aim of Responsible Innovation (Stilgoe et al. 2013a, b) (or ‘RI’) which has in turn been used to try to ‘flesh out’ RI in the context of health and biomedical innovation (Lipworth and Axler 2016). Similarly, Fraaije and Flipse (2020) describe RRI as gaining momentum, but lacking collective meaning, and therefore being described in an ad hoc fashion in the literature. They also present a framework for implementing RRI, in their case synthesised from their literature review.

RRI has been described as evolving from considerations of ethical, legal and social implications of research (Owen et al. 2012); and the ‘responsiveness’ which forms part of RI frameworks is often to the very same ethical, legal and social issues which permeate innovation regardless of the difference in terminology. First popularised by the Human Genome Project, the term ‘ELSI’ attracted some controversy among science and technology researchers, particularly around the alleged division of labour ‘ELSI’ dedicated work packages can create. Allocation of ethical, legal and social considerations to a set group of individuals within research consortia potentially removes such thinking from the leadership, thus marginalising the very issues it seeks to promote. Discussions of RRI, by contrast, have emphasised that this is not a peripheral task to be delegated to ethicists or social scientists, but rather a joint responsibility on scientists, universities, innovators and funders to change the processes through which innovation is delivered (Owen et al. 2012).

If ‘ELSI’ scholars (lawyers, ethicists and social scientists) are less marginalised within ‘RRI’ approaches, and do not function as the lone conscience of innovation, this is certainly welcome. This will not necessarily resolve all of the governance ‘cracks’ along which research consortia can split: at organisational, sectoral and national levels (Wallace 2011). However, if the organisations which fund and make up consortia can be united in a shared sense of responsibility to society at large in their contributions to innovation, this could help overcome key challenges in the implementation of RRI within the governance frameworks of research consortia.

The contribution of this paper is therefore not to present a further general framework for RRI implementation, as these have already been posited in and from the literature. Instead, given the importance of scientific consortia for innovation, we consider RRI implementation within the particular context of the governance of research collaboration, bearing in mind the conventional institutional arrangements which are reached as part of these undertakings, and the challenges and limitations these structures can create.

## The governance of consortia

‘Governance’ is a term used in many contexts and with many connotations. There is no universally agreed definition, especially as ‘governance’ is often used interchangeably with ‘regulation’ or even ‘regulatory governance’. Nonetheless, there are some widely agreed components. Governance involves attempts to constrain and direct behaviour, not only of individuals, but also at the group level (Brownsword and Goodwin 2012). Moreover, such attempts must be systematic and purposeful, involving ‘the sustained and focused attempt to alter the behaviour of others according to standards or goals with the intention of producing a broadly identified outcome or outcomes’ (Black 2002). Good governance need not be top-down and hierarchical, but may be enacted by many stakeholders, including those who are themselves governed: ‘regulatees’ also construct the governance setting applicable to them’ (Harmon 2018). As this suggests, the means or instruments of governance are hardly limited to national legislation, but include a more diverse and flexible range of mechanisms such as professional codes of conduct, market incentives, good practice guidelines, technical standards, and performance evaluation (Ayers and Braithwaite 1992).

Here, we are concerned with governance within large research consortia. For research consortia, governance systems comprise people and groups (state-constituted regulators, funders, institutions and individual actors); policy (e.g. law such as the GDPR, which we will consider in this paper) and processes (shared culture, institutional procedures, values and norms) (Kaye et al. 2012) operating at the macro (national, regional), meso (sectoral, institutional) and micro (professional, individual) levels (the 3 M’s) (WHO 2002). The purpose of consortia governance and its ‘broadly identified outcome or outcomes’ is to enable the project’s key activities to be carried out in a way that balances the interests of the different consortia partners in an equitable manner. The opportunities and challenges of consortia, and the reasons why they can be seen to require a governance framework are discussed in more detail below.

RRI now plays a part in constituting the governance systems of many consortia (and is mandatory for projects funded through the European Commission) (Harmon 2018). RRI principles add an additional layer of functional requirements to the traditional aims and desired outcomes of research consortia: legitimate science and innovation activities must now also focus on achieving public goods, particularly sustainable social and environmental benefits; be assessed in part on how well they facilitate positive social, ethical and environmental impacts; and integrate ongoing dialogue with communities of interest, including public and non-governmental organisations (Sutcliffe 2011). Well-functioning research practices therefore need to be embedded into governance structures and routines that value and enable RRI principles. Such governance structures must in turn be flexible and capable of adapting to change, with all stakeholders in a research consortia aware of the aims, objectives and expected impacts of the governance structures in order for them to be effective.

The governance framework of each consortium is to an extent unique to that research consortia as a result of the specific institutions, sectors, policies, and jurisdictions it encompasses. However, the contractual underpinnings will be inherited from the standard templates provided by the funder, and often (as revealed in the authors’ qualitative discussions) modified by institutional representatives, with the (commercial) interests of the contracting parties in mind. Nonetheless, the common aspiration of each particular governance system is to produce a set of mutually agreed, shared requirements and operational responsibilities that act as a framework for ensuring the legitimacy of decisions made in pursuit of key project aims, including meeting its RRI goals.

## Why is governance needed in research consortia?

Increasing scientific specialisation, coupled with the growing need to address complex, multi-faceted challenges such as climate change or translational research, means funding agencies are increasingly promoting multi-disciplinary project teams. Life scientists, for example, increasingly need to work with computer scientists to manage large, digitised data sets such as whole genome sequences; as well as with lawyers and ethicists to address the complexities of recruiting and collecting data from human participants. As a result, project teams are not only getting larger, they are also becoming more geographically dispersed, incorporating researchers from multiple different institutions in different countries and different cities.

A further level of complexity is added by the growing popularity of public-private partnerships that bring together academic centres with commercial companies, often with the aim of creating large, communal resources of materials and data (Altshuler et al. 2010; Lim 2014). Companies have their own management structures, reward systems and mechanisms for extracting value from scientific research such as patents, which may be difficult to align with academic goals (Evans 2010; Morandi 2013). It has even been suggested that the ‘traditional’ paradigm of ‘open science’ based on free collaboration and unrestricted disclosure of results is being replaced with that of ‘open innovation,’ characterised by exclusive ‘partnering’ relationships (EUA 2005).

Such large, multi-national, multi-institutional and multi-disciplinary groupings increase the potential for conflicts about co-ordination and organisation of the work, allocation of resources and workloads, assignment of credit and authority within the project, different management styles, and what researchers in different fields, different countries or at different levels of seniority hope to get out of the collaboration (Hackett 2005; Shrum et al. 2001). Companies working with academic scientists almost always require formal contractual measures to govern the ownership and distribution of intellectual property rights arising from the work, while contractual arrangements can provide reassuring safeguards for academic researchers working with unfamiliar new colleagues (Morrison 2017).

The scale of collaboration and the need for data sharing increasingly require European science to operate through consortia, with formal management structures, reporting requirements, and written rules and regulations to cover areas of responsibility from data ownership to research dissemination. These collaborative arrangements are more complex and open-ended than simple contract research (EUA 2005), and almost always require contracts to balance the interests of the various parties (DESCA 2017). The focus of such agreements, however, is mainly to achieve an acceptable level of equity between the partners, rather than to consider the interests of broader groups of stakeholders across society, such as patients or data subjects (Harwich and Lasko-Skinner 2018).

In particular, the volume, scale and complexity of data generated in a contemporary science project can become a source of tension over ensuring all partners have equitable and timely access. Big Data are, by common definition, heterogeneous in origin and not necessarily designed for interoperability - so much so, that Horizon 2020 multi-disciplinary consortia have been funded specifically to address the governance challenges of data integration (EU-STANDS4PM n.d.). Having a governance system can be a way of ensuring that there are clear rules about how data access, publication, patenting and other consortia activities will operate; and it delineates responsibility. Having transparent and agreed procedures and responsibilities before a project commences acts as a way of defusing potential conflicts before they arise; and for dealing with them in an appropriate and fair fashion if they do occur (Budin-Ljøsne et al. 2014; Kaye and Hawkins 2014; Morrison et al. 2015; Muddyman et al. 2013; Teare et al. 2018).

## Forms of consortia governance

Every international consortia will be set up differently depending on its aim, but it is expected that there will be common elements. In fact, for reasons explained below, it can be these ‘boilerplate’ commonalities that hamper the broader RRI focus of project governance. The primary influences will be the project funders; the institutions involved in the research consortia, which are bonded together by means of a contract (the main legal mechanism that directs the consortia); and the project leadership and management. The DESCA 2020 model consortium agreement (DESCA 2017) provides a good illustration of how these structures are conventionally embedded within contracts.

By placing these influences in chronological order, it is possible to understand their respective impact on a consortium. The funder will inevitably offer an award on their own terms, giving them a significant role in shaping the initial foundations of project governance. A grant agreement is likely to confirm not only the timescales for delivering the project, but also reporting periods and mechanisms, the costs eligible for reimbursement and the party within the project responsible for managing the funding, who correspondingly assumes a central role within consortia. The terms of the funding may also include stipulations as to how data generated under the project are to be put to further use through archival or open access arrangements. Whatever the precise terms, the overarching effect is that project governance cannot be designed from a blank slate, as its contractual genesis can be traced back to the requirements of the funder.

Assuming the parties to a consortium agree to the terms of their funding (they may have a limited scope to negotiate), they must also agree the terms of their collaboration amongst themselves. A consortium or project agreement between the parties must be negotiated to assign and protect intellectual property, as well as assigning rights and responsibilities between partners, and addressing liability for external (or even internal) claims. In some cases, such as the European Commission’s ‘Innovative Medicines Initiative’ funding stream for public-private partnerships in drug discovery, the funding agency, here the EC, acts as a ‘neutral broker’ overseeing contractual agreements between consortium partners, further extending the funder’s influence (Goldman 2013).

Assembling a project contract may, again, be a banal process for researchers, but it is a stage in which the blueprint for much of the significant decision-making in the project can be set out. Even if the project’s decision-making processes *are* of interest to its prospective researchers, they may play a limited role in contractual negotiations by their institution. Innovative elements of consortia governance can subsequently be more difficult to introduce if they do not have the support and authority of the contractual underpinnings of the project (see Fig. 1 below). For example, a data access committee may be set up, supported by a criteria set out in a published policy, into which key stakeholders have input. However, if the contract gives parties ownership of any new knowledge or data they generate (sometimes referred to as ‘foreground’ in contractual terms) and corresponding rights to grant or refuse access to that data without reference to the decisions of such a committee, the authority of this open, transparent feature has already been undermined by the contractual arrangements of the consortium.

By the time project management and leadership comes into play, much of the structures that organise the running of the project (a large part of the project governance) will have been shaped by contract. Whether built into a contract, or designed subsequently by researchers, the conventional governance frameworks will typically comprise elements of:

- Management - a management team or steering committee responsible for the overall management of the research consortia. Often it will be located at, led by and populated by staff at the institution of the principal investigator where the grant funding was obtained.
- Advisory Board - experts in their field and independent of the research consortium, who meet regularly (usually annually or bi-annually).
- Contributors - consortia participants who provide research findings, raw data, new technologies or other elements needed to advance the work of the research consortium.
- Committees - focussing on specific aspects of the research consortium’s work, which may be in place for part, or for the duration of the project funding (for example, assisting with formation of the consortium or project deliverables).

A generic governance structure is shown in Fig. 1.

### Key

C1-C3 indicate such committees as may be appointed by the management team to deal with specific issues, for example: a data access committee; a publication committee, where academic partners are involved; or an ethics committee, to provide ethical input as the project work proceeds. These are not elements of consortia governance which will necessarily be implemented in every case, but some projects have been more innovative in introducing committees with clear, transparent policies for decision-making, for example in relation to publications and data access. (Morrison et al. 2015; Teare et al. 2018).

The external governance framework consists of those national and international regulatory agencies, statutory laws, Research Ethics Committees, codes of conduct, best practice recommendations, guidelines, institutional rules, terms of employment, and similar components. That is, the ‘macro’ level of governance that typically pre-exists any particular consortia and must be taken into account when assembling the meso and mirco level contractual agreements that enact the consortium and the more temporary internal project governance structures, such as the management group (Kaye et al. 2015).

Project governance can be conflated with co-ordination and management activities. Governance is the attempt to direct behaviour in a systematic way. In consortia, governance can be helpfully understood as the structures and rules that prescribe and direct actions, while management consists of the decision making that takes place within the scope of those rules and structures. By way of illustration, in Fig. 1 above, the ‘management team’ box represents the fact that there is a designated management group and the terms under which that group operates. These terms may be codified in the funding arrangement, project contract or elsewhere, but they must stipulate roles and responsibilities of management team members. The same applies to whatever committees (C1-C3 in Fig. 1) are created during the lifetime of the project. Project management, by contrast, is not the existence or terms of the management group, but the actions and decisions taken by its members over the duration of the consortium. Governance and management are thus closely related: for example, the management team may take a decision - as long as the power to do so is stipulated in their terms of reference - to create a new committee to address a particular issue facing the project, thus modifying the governance structure of the consortium. Nonetheless, the distinction is important. Failing to distinguish between the two entities can limit the ability of those charged with governance to design adequate governance structures prior to implementation, by encouraging their development in a piecemeal fashion alongside management tasks; whilst confusion around roles and responsibilities can erode management coherence. Having adequately distinguished the tasks of management and governance, the next section sets out four significant challenges in using conventional governance structures for large research consortia.

## What are the challenges of implementing RRI in conventional research consortia?

Experience and critical analysis have permitted us to tease out some of the main challenges that were encountered during our involvement in conventional consortia governance. This experience has provided practical evidence that they tend not to lend themselves well to the implementation of RRI. Here we identify and highlight four features of consortia governance that pose non-trivial challenges for implementing RRI in research consortia governance, together with suggested recommendations for meeting them:

- Governance lock-in and effective autonomy:

The first challenge to the implementation of RRI within the governance framework of a research consortia is that the contractual agreements founding the project are often drawn up from standard templates with limited researcher involvement. This means that, even where researchers do want to implement ‘values-sensitive design’ (Van den Hoven et al. 2012) in the project governance, the values reflected in this contractual model will be those of the funder, combined with those of the respective consortium institutions. The representatives of said institutions may, for example, value the allocation and preservation of intellectual property over and above the values of ‘open science,’ given that their role is to promote the commercial interests of their clients or employers. ‘Responsible’ researchers may wish to implement a data access committee, for example, which could distribute information in order to promote the ‘right impacts’ of science, but they will be unable to do so unless institutional data owners have granted access rights for these purposes within the initial agreement. The potential exclusion of researcher aims and values from the contractual basis of the consortia is a key initial challenge for implementing RRI in the consortium, as it can then inhibit researchers from implementing subsequent aspects of responsible research.

Effective governance therefore requires forward planning. It must be shaped by a workable model devised from the outset of the project; monitored; informed by and responsive to scientific and contextual developments; and accessible to all consortia members as and when required. However, a consortium is almost never a legal entity in its own right, and it is therefore reliant on contractual implementation from the institutions in which investigators are located for key decisions, such as an external transfer of data or other intellectual property. Unless the autonomy for one or more bodies within the consortium to make decisions with potential legal implications (for example, about access to consortia-generated data) has been built into the consortium design and ‘signed off’ by the institutions in the initiating consortium agreement, decisions made at project level may prove to be largely conjectural. Modifications may be possible through negotiations in individual cases, but updating funder template contracts to ensure resources and flexibility to implement (for example) public engagement as part of RRI would be a very attractive solution, which would help embed RRI in the foundations of the consortium.

- Public or patient participation and integration:

The idea of public engagement and involvement with research is increasingly promoted as desirable. Stilgoe et al. (2013a, b) argue for the democratisation of science in which transitioning from ‘monologues to dialogues’ with the public can positively impact on institutional policies and practices. This is especially the case for patients taking part in medical research (Gregory et al. 2018) and where research utilises data derived from or about human participants (Shah et al. 2019a; Shah et al. 2019b; Kaye et al. 2018). However, the inclusion of meaningful public or patient participation in consortia represents our second challenge for conventional governance systems. At an institutional level there are already a large number of requirements (such as employment requirements, intellectual property protections and material transfer agreements) which will have an effect on the governance processes that are put in place. Funders also make their own requirements that influence the activities of consortia, such as data sharing and open access policies. This web of interests which *must* be accommodated is already so complex that the voluntary introduction of an additional factor, such as participator co-design of research or inclusion of public or patient representatives in project governance bodies, can be a challenging prospect.

Whilst development of patient and/or public involvement (PPI) activities can be iterative throughout the duration of any project, consortia would benefit from early planning about what the project needs to communicate to participants, why and where the participants need to be engaged and involved, and how this can be achieved. Consortia need to build structures of inclusion, as well as support the researchers who champion engagement and involvement.

The drive of research funders towards greater citizen engagement and involvement in research should be also met with a commensurate shift in the way the integration of PPI and communication strategies are assessed. Inclusion of involvement and engagement professionals during the grant review processes should be matched by the inclusion of ethics, law and social science professionals, who can ensure academic rigor in governance plans. The key question consortia need to ask is: ‘Who is speaking for the participants’ interests?’ Ethicists, lawyers and social science researchers are best placed to guide how those interests can inform project governance and shape policy, whilst involvement and engagement professionals can critically review the feasibility of PPI plans, and inform their design.

Moreover, overly rigid governance structures can limit the scope for consortia members to make any practical accommodation for the views and preferences of lay participants, leaving engagement as a tokenistic exercise or a one-way ‘deficit model’ of provision of information (Simis et al. 2016; Hartley et al. 2019), rather than a dialogue with participants. A further aspect of this challenge is that legal liability (such as compliance with data protection law) tends to lie with the institutions, rather than individual researchers involved in the consortium. Involving lay participants in decisions about the collection or use of personal data (including transfer to third parties) could expose them to the same legal liabilities as the consortium institutions, but without the institutional resources and finances to bear the cost of any infringement proceedings. This is not only unfair and potentially harmful to lay participants, but it also acts as an obstacle to encouraging or enabling their participation in shared decision making.

We agree that integration of PPI within a research endeavour is one mechanism to explore social implications and acceptability of a subject, but for any such initiatives to be more than tokenistic, a number of elements are required. For example: funding researcher time to strategize and implement involvement and outreach activities; clarity that ‘lay’ participants taking part in project governance are eligible to claim costs, in the same way as academic participants; confirmation that decisions relating to project intellectual property (such as data and samples) can be made on a joint or delegated basis, even including the input of third party, lay participants.

At the same time, however, reasonable parameters need to be established. There is increasing recognition of research participants as ‘collaborators’ in the development of research projects (Gregory et al. 2018). But legal status as a collaborator within a consortia brings with it burdens, which research participants should not share. For example, if a patient representative helps to determine how project personal data are used, there should be clarity that in doing so this individual does not become a joint data controller with the consortia parties, with all the attendant liability under data protection law. There is thus an inevitable tension in sharing the ‘benefit’ of controlling innovation with ‘lay’ participants, without also sharing the corresponding burden of taking legal responsibility for said control. For all that project co-design is desirable, RRI advocates must be realistic about its limits. Consortia parties retain ultimate control of legal obligations for the very valid reason that the alternative would mean sharing legal exposure with private individuals. Co-design has to operate within responsible parameters - even if it may seem ‘undemocratic’, appropriately resourced institutions should shoulder the financial risks of innovation. They will therefore require sufficient control to ensure consortia decisions do not trigger this potential liability.

Achievement of meaningful public or patient engagement in research studies should result in participants feeling ‘empowered’, without the burden of associated liabilities and responsibilities which fall on those party to the research contract. However, this desirable RRI goal presents a very real structural impediment, because of the difficulty posed in trying to give data subjects’ power without liability; and it represents a governance reason for why this kind of empowerment does not work. Alternative roles for research participants, for example in the shape of patient advisory board members or survey participants, has the advantage of encouraging participation in a much more inclusive way. This positive use of research participants would allow them to perform a role that is incorporated into the consortium governance, without the potential negative burden of them committing any legal breach on behalf of the institution(s) involved (or making them responsible for the discharge of any legal obligations). The consequential meaningful engagement would also present an opportunity for participants to be rewarded for their involvement in the research project.

This second challenge to implementing RRI can therefore be summarised as follows: not only must contractual foundations allow for the operation of RRI principles, but governance structures must be ‘democratised’ within realistic limits. To a degree, that enables participants’ genuine influence over the research consortium, without eroding institutional responsibility for their legal obligations. This is in turn relevant to the third governance challenge for responsible research: implementing effective transparency.

- Transparency:

Transparency has long been acknowledged at academic and policy levels as a core feature of an RRI process (von Schomberg 2011). However, the exclusive, licence-orientated ‘partnership’ model of open innovation, as opposed to open science (EUA 2005) is intrinsically less transparent than the free, unrestricted sharing of data envisaged by open science. Measures to counteract the potential for opacity in consortia innovation are therefore needed for effective RRI implementation. For the purposes of this paper, where we are concerned with the legal as well as the broader aspects of consortia governance, it is interesting that two potential meanings of transparency can emerge from the literature.

Firstly, in the context of governance, ‘transparency’ refers to the accessibility and visibility of the governance structures of consortia. Good governance requires that those internal and external to the project know what governance structures and procedures are in place; what mechanisms for legitimate decision-making have been adopted (for example, whether all consortia members vote on key decisions, or whether there is a more limited forum of representatives from each institution or research group, or something else); and where authority and responsibility for different types of actions are located in the consortium. This includes communicating what mechanisms are in place for contesting decisions, raising objections or concerns, seeking redress, or proposing changes. RRI also requires that the consortia’s broader purpose and the public good it is intended to achieve are communicated in comprehensible language(s) to those external to the project, including lay audiences (Harmon 2018).

Secondly, and more specifically, transparency is one of the key principles of data protection introduced by the GDPR in the European Economic Area. Where personal data are collected from a subject (or obtained via a third party) the controller of that information must provide this individual with a mandatory list of information including who will access their information, and for what purposes. This information must be accessible and meaningful. Transparency is not a one-off exercise in communication, but must be actively updated whenever there is a material change in processing which might impact upon subjects’ fundamental rights (Article 29 Working Party 2018). This in turn aligns with the broader requirement to act in accordance with individual’s reasonable expectation of privacy, wherever private or confidential information are used (Taylor and Wilson 2019). Such flexible, engaged and transparent models are important for ensuring that there is no improper severance between people and the information which relates to their lives or identity (Ballantyne 2020).

There is a connection between transparency as relating to the visibility of a governance structure and to the GDPR’s requirements. A visible, accessible governance structure will naturally assist in providing clarity to data subjects as to who is accessing their data and (ideally) according to what criteria. A published data access policy is particularly helpful in this regard (Teare et al. 2018), especially if it is actively drawn to the attention of the data subjects who might be interested in the terms on which their information is granted access. As discussed earlier, this is much more desirable than simply relying on contractual clauses which allow intellectual property owners to grant access at their discretion, with no checks on the potential opacity of such decision-making. Additionally, where consortia members jointly determine the means and purposes of data processing, they are required to make transparent arrangements as to their respective responsibilities for ensuring GDPR compliance of such processing. This is yet another reason why clear, visible consortia structures are important from a legal perspective, as well as for implementing RRI.

However, achieving visible infrastructure and GDPR compliance is not easy. Meaningful transparency in the context of governance can often be tokenistic. Websites that lack ‘the detail required to emphatically meet the demands of RRI’ (Harmon 2018:25) are likely to provide limited accessibility and little scope for interactive communication. In addition, external regulatory bodies may have standard templates for communicating information to external audiences (and/or research participants) which they provide as evidence of good governance; but these templates may not be compatible with either the needs of the consortium or with the requirements of other elements of the external governance environment, such as data protection law. If meaningful involvement and engagement of research participants has been achieved, lay representatives can also be utilised as a sounding board for transparency. Even where it is not possible to achieve project co-design with research participants, transparency can be used to promote and ensure that accessible and visible governance structures are in place.

As with participant engagement, a key element to overcome the challenge of communicating the research consortia’s purpose and public benefit in easily comprehensible language, is to ensure that there is sufficient budget and human resource time allocated to developing, testing and delivering the means by which the consortium’s purpose and governance structure are made accessible. This should include the use of research documentation that is easy to read and to understand; with the right balance being struck between candour and reassurance. The enhanced transparency obligations placed upon data controllers by the GDPR require accessible and coherent information to be provided to data subjects. Researchers embedded in their field may not always be the best judge of accessible information; but well-integrated lay representatives are a good sounding board for transparency of information over the life-time of the project. If, ideally, these representatives are drawn from the participant population, they may well reflect the level of literacy participants will have vis-a-vis their own situation (for example a specific occupation, or people with a particular condition), but also be able to highlight where scientific or legal information strays beyond language meaningful to them, and requires more explanation.

- Legacy planning:

Our final challenge of implementing RRI in research consortia looks towards the future and relates to consortia governance at the end of the research project. Consortia are necessarily time-limited endeavours. Funding agencies typically provide only fixed-term financial support, with endpoints and deliverables often contractually mandated. Consortia governance arrangements are similarly durational; they are typically ‘established for a limited duration and purpose, and [ …] dismantled once the project ends and it is no longer needed’ (Kaye et al. 2015). However, as consortia are increasingly funded to build and curate large, heterogeneous data sets (which may include newly generated data, aggregations of existing data held by partner institutions including public bodies, and annotations and metadata relating to the pooled data), consortia must increasingly deal with the issue of how these data sets are to be governed once the consortium ends.

‘Data intensive’ or ‘data-driven’ approaches to the life sciences envisage, and require, new roles and responsibilities for scientists, new infrastructures and new governance arrangements (Swierstra and Efstathiou 2020; Leonelli 2012). The great promise of large datasets is that they can be re-used over time by the wider scientific community to address new questions and attain insights unavailable with traditional datasets. However, this requires that the data be readily accessible, that the datasets themselves are curated and maintained, and that active governance is sustained (Wilkinson et al. 2016; Stuermer et al. 2017). Much life sciences data still requires manual curation, which is time consuming and expensive (Leonelli 2016). Data curation and governance is ongoing, not a one-off task, and many repositories require financial support to sustain them (Stuermer et al. 2017). Sustainability of data resources is thus a global challenge for the life sciences, as the case of biobanks has illustrated (Chalmers et al. 2016). In this context the challenge of making legacy arrangements for large consortia can be viewed as a specific manifestation of a larger and more pervasive problem.

The transitory nature of consortia governance and the above-noted fact that consortia rarely have legal personality, means that once the project ends and the contractual arrangements supporting it expire, the data access provisions and rules agreed during the lifetime of the consortium no longer hold. In the worst-case scenario, this leaves a vacuum with no clarity over who has authority over, or ownership of, the data. More likely, control of datasets will default to the lead institutions of the former consortium, or to those which originally generated each particular piece of data. This can lead to fragmentation of aggregated ‘big data’ sets as different owners may not agree compatible access provisions after the end of the consortium; for example, one institution may favour open access, while another may wish to deposit a portion of their data in a for-profit repository, or protect it to secure future IP claims. This, in turn, can lead to underuse of the very kinds of large data sets that consortia were funded to produce in the first place. The dismantling of consortia governance structures at the end of the project also reduces the capacity of consortia to enact and enforce input from participants and other public bodies about how the data (often derived from volunteer research subjects) should be used and handled after the lifetime of the consortium. This poses yet another threat to meaningful engagement.

Lessons learned from the research participant collaborators during the lifespan of the project should be used to inform legacy planning for the end of the project. Whilst it is not possible to anticipate all of the issues that a consortium will encounter, issues that require a change in the governance structure will undoubtedly arise as the research progresses: for example, questions about who has responsibility for co-created data and samples when the project ends and funding ceases, will become pertinent issues over time which might not be afforded sufficient attention at project outset. In situations such as this, where research is required that was not anticipated at project outset, there should be flexibility to reallocate resources across the consortia to provide additional capacity as required, so as not to detract from other research or monitoring tasks.

We contend that if the key governance challenges set out above are not effectively overcome, then successful implementation of RRI in a research consortium cannot be fully achieved. Once achieved, however, incorporating RRI considerations into consortia is not intrinsically problematic, but will be ineffective if consultation with legal and ethical coordinators is not a required part of major decision-making.

**Fig. 2:**
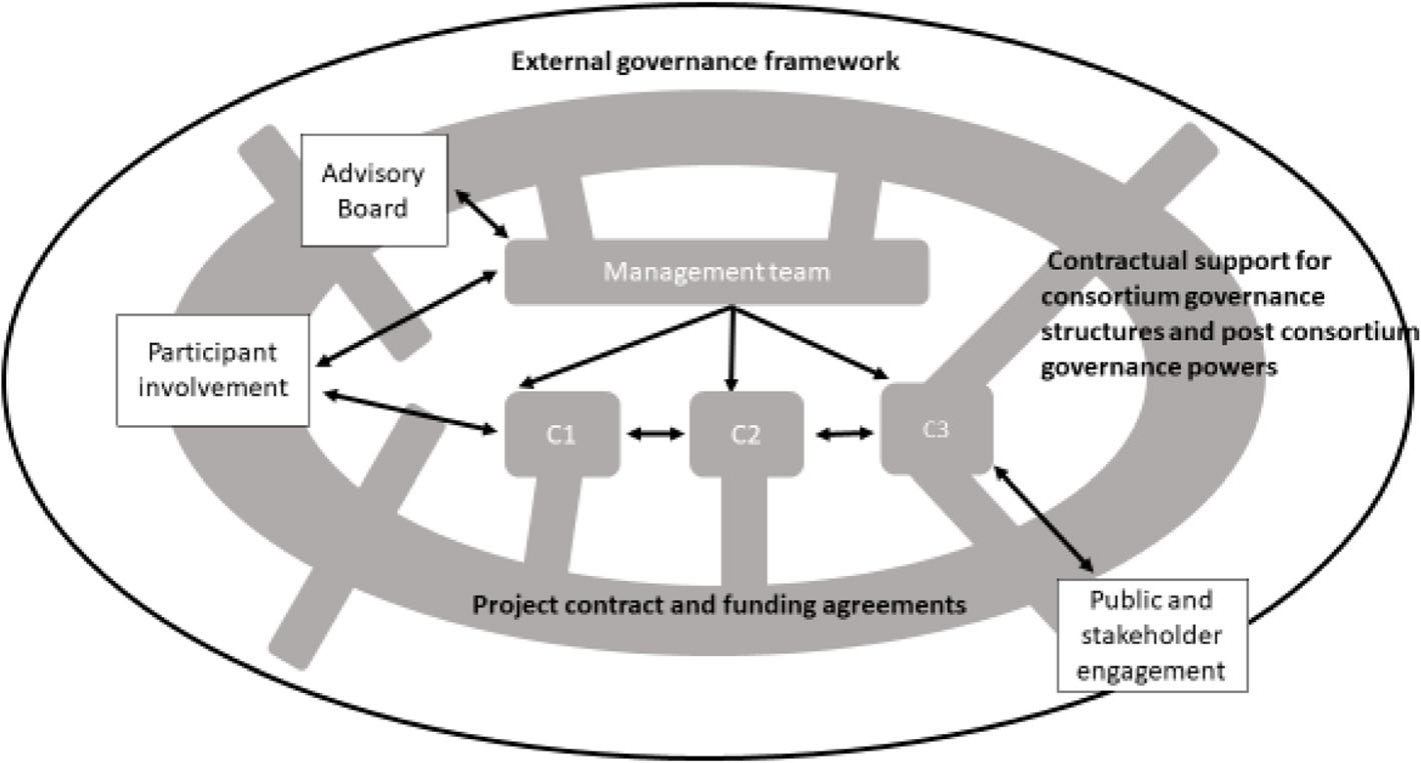
Improved integration of Governance facilitates Responsible Research and Innovation by incorporating community and our other recommendations

An example of how RRI can be incorporated into the architecture of a consortium is set out in Fig. 2. Centralisation of ELSI principles from the start of the project puts a consortia in a better position to react to any changes in the law, or public opinion, over the course of its lifespan; and a more integrated team will be better placed to explore the legal, social and ethical dimensions of responsible innovation. In order to achieve this, the contract between funding body and consortium institutions must contain provisions to embed and enable RRI activities such as the capacity to make decisions about future ownership, uses and storage of data that have legal foundation during and after the lifetime of the project itself. Critically, as the remit of potential data uses become increasingly stretched, it must also be recognised that designing effective governance is a research task in its own right, involving complex analysis of law, regulation and ethical underpinnings which will continue to evolve throughout the project lifespan and beyond. Hence in Fig. 2. the research contract is anchored in the broader external governance framework, the better to support and sustain the internal governance structures of the consortium. Furthermore, enabling researchers in ethics and social sciences to monitor scientific developments allows them to identify where research is challenging the boundaries of acceptable or anticipated practice, or raising new questions. This primary research needs to be recognised by constituents from other collaborating disciplines, sufficiently resourced, and valued as a project outcome.

Once RRI is successfully implemented into a project, it is important to maintain this work throughout the lifecycle of the project. As a project progresses, governance systems, as with all components of the research, will need to be reviewed regularly; and the opportunity for their frameworks to evolve must be in-built to accommodate revision over time.

## Conclusion

In this paper, we have identified consortia as the predominant organisational form through which European scientific research is conducted, but one which poses particular challenges to the effective implementation of RRI measures and activities. Large consortia, spanning different institutions, jurisdictions, and increasingly involving public and private sector actors working collaboratively, require governance structures to ensure their lawful and efficient operation. We have discussed how typical governance arrangements for research consortia are structured by a mixture of funding agency requirements, the policies and practices of the institutions employing the researchers taking part in the consortium, and an array of existing regulatory requirements from employment, data protection and health and safety laws to rules governing the participation of human subjects in research. Many of these requirements manifest in the formal contract between funding providers and recipients.

Broadly, the RRI agenda was devised with the intention of making research more responsive to both societal needs and social concerns about science and innovation. At its heart is a normative idea of responsiveness and adaptability: research and innovation should be inclusive, responsive and, ideally, tailored to the needs of ‘end users’ while recognising that these will often be heterogeneous and local in nature. This envisages innovation as a two-way dialogue between researchers and citizens or participants. Practical RRI measures could range from participant participation on a data sharing committee or patient-led co-design of outcome measures, to lay feedback on the comprehensibility of an information sheet. In theory the requirements of RRI can and should be supported by appropriate consortia governance mechanisms, such as having a data sharing panel with terms of reference that allow lay participation, or having the mechanisms that allow public consultation and feedback into formal decision making by the consortia management.

However, as we have illustrated above, this requirement for flexibility and inclusion is often at odds with the rather rigid terms and conditions imposed on consortia by contractual and institutional requirements. This process begins with the terms of funding and the drafting of contractual terms, which underpin the consortium. Although researchers write the grant proposals, they generally have less input into the contracts that enact the successful grants. This can limit a consortium’s decision making capacity, and thus its ability to support a governance framework that enables adaptation and response to public input, from the beginning. We suggest that researcher involvement in the formulation of the aims and values of the initial contract should be considered a prerequisite for effectively implementing RRI in research consortia governance; as well as presenting an opportunity to exploit an initial window for implementing RRI structures. However, this would represent initial unpaid work by researchers outside their normal areas of expertise. Therefore making contracts more flexible and enabling of RRI measures also requires greater efforts from funding agencies and institutions taking part in the consortium, who typically delegate contract negotiations to in-house specialist administrators In this instance, researchers are to an extent the ‘lay’ stakeholders seeking input to another groups’ expert domain.

This initial lack of capacity for flexibility and responsiveness is compounded by the way in which research projects are envisaged as fixed term endeavours with a pre-determined, often pre-allocated budget and set outcomes (deliverables’) agreed in advance. This, plus an emphasis on maximising speed and minimising cost, puts real pressures on any consortium trying to build in outreach and the ability to meaningfully change goals, or means, in response to stakeholder feedback during the lifetime of the project.

This is not to say that implementing RRI necessarily slows down research, or makes innovation unduly expensive. Truly two-way engagement outside the research consortium requires sufficient agility to adapt to feedback; the consortium must be sufficiently responsive to be able to react to the outcome of external engagement exercises. This would suggest (for example) that consortia management processes should not be so minutely prescribed in project agreements that they cannot evolve to respond to feedback, or include new voices where appropriate. As for expense, it has been noted that regulators often carry the burden of gaining public acceptance for innovative products, potentially at the cost of simplifying risk (Stirling 2017). Upstreaming the responsibility for engaging public acceptance to research funders lessens the risk of pouring money into an innovation which, ultimately, a regulator cannot licence because it would involve risk the public will not accept.

As consortia rarely have legal personality, attempts at involvement can also be limited by measures in the external governance framework that would expose participants to liabilities as individuals without the protection of institutional status and resources that most official consortia partners are afforded. As a result, RRI can default to engagement-by-dissemination, and engagement can default to one-way information provision rather than dialogue. Thus even when researchers have a genuine desire to implement RRI, input from the public and ELSI researchers alike can become instrumental and tokenistic, rather than genuine and response driven as a result of governance lock-in, pressures of time and finance, and external governance requirements. Finally, the fixed term duration of consortia increasingly funded to create lasting resources of materials and data, means that any decisions made by the consortium, no matter how engaged and involved, rarely have any influence beyond the end of the consortium itself. After the end of the funding period, the products of the consortium tend to default to institutions, which may also have interests that do not align with the goals of RRI, or may lack capacity to incorporate RRI measures.

All of these elements, individually, but even more so in concert, act as impediments to consortia governance frameworks that can support, rather than inhibit, RRI. The way forward, we suggest, requires more recognition of this problem by funders and research institutions (especially universities). We do recommend that researchers have more input into contract provisions to ensure these support RRI, but the broader set of issues identified here cannot be addressed by simply giving researchers even more responsibilities. Rather, funders must do more work to ensure their different priorities such as open data, value for money, and timely project completion are in alignment with, and ideally subordinate to, RRI. Institutions and funding agencies should work together to design contracts for consortia that recognise the challenges of RRI and provide support for consortia governance capacities to actively implement and respond to RRI input. These also need to recognise where elements of the existing external governance framework can inhibit RRI activities and incorporate powers and functions for consortia - at the planning stage - that can ameliorate these problems. For example, if there are good reasons to have lay participation in a data management committee, then the lay participants need to be shielded in some way from liability as data processors; and the capacity needs to exist for their decisions, and potentially their activities, to persist after the official end of the project. Clearly these are areas where more work on the appropriate financial and legal mechanisms needs to be undertaken, but we hope that our modest contribution can initiate these bigger conversations and efforts.

## Data Availability

No datasets were generated or analysed for this manuscript.

## Abbreviations

EC: European Commission
ELSI: Ethical, Legal and Social Issues
GDPR: General Data Protection Regulation
PPI: Patient and/or Public Involvement
RRI: Responsible Research and Innovation

## Footnotes

1 A cross-cutting issue in Horizon 2020, the EU Programme for Research and Innovation 2014–2020
